# Morphological Aspects and Viremia Analysis of BALB/c Murine Model Experimentally Infected with Dengue Virus Serotype 4

**DOI:** 10.3390/v13101954

**Published:** 2021-09-29

**Authors:** Arthur da Costa Rasinhas, Fernanda Cunha Jácome, Gabriela Cardoso Caldas, Ana Luisa Teixeira de Almeida, Marcos Alexandre Nunes da Silva, Daniel Dias Coutinho de Souza, Amanda Carlos Paulino, Derick Mendes Bandeira, Raphael Leonardo, Priscila Conrado Guerra Nunes, Ronaldo Mohana-Borges, Ortrud Monika Barth, Flavia Barreto dos Santos, Debora Ferreira Barreto Vieira

**Affiliations:** 1 Laboratory of Viral Morphology and Morphogenesis, Oswaldo Cruz Institute, Fiocruz, Rio de Janeiro 21040-900, RJ, Brazil; fernandacunhajacome@gmail.com (F.C.J.); gabrielacardosocaldas@gmail.com (G.C.C.); analuisaprovoc2012@gmail.com (A.L.T.d.A.); marquinhosans@hotmail.com (M.A.N.d.S.); daniel.brasil11@hotmail.com (D.D.C.d.S.); amandacarlos.bio@gmail.com (A.C.P.); derick_mendes@live.com (D.M.B.); raphabala28@gmail.com (R.L.); monikabarth@gmail.com (O.M.B.); barreto@ioc.fiocruz.br (D.F.B.V.); 2 Laboratory of Viral Immunology, Oswaldo Cruz Institute, Fiocruz, Rio de Janeiro 21040-900, RJ, Brazil; pricgn@ioc.fiocruz.br (P.C.G.N.); flaviabarretod1@gmail.com (F.B.d.S.); 3 Laboratory of Biotechnology and Structural Bioengineering, Biophysics Institute Carlos Chagas Filho, Rio de Janeiro Federal University, Rio de Janeiro 21941-901, RJ, Brazil; mohana@biof.ufrj.br

**Keywords:** DENV-4, BALB/c mice, liver, lung, heart, histopathology, ultrastructure

## Abstract

Ever since its brief introduction in the Brazilian territory in 1981, dengue virus serotype 4 (DENV-4) remained absent from the national epidemiological scenario for almost 25 years. The emergence of DENV-4 in 2010 resulted in epidemics in most Brazilian states. DENV-4, however, remains one of the least studied among the four DENV serotypes. Despite being known as a mild serotype, DENV-4 is associated with severe cases and deaths and deserves to be investigated; however, the lack of suitable experimental animal models is a limiting factor for pathogenesis studies. Here, we aimed to investigate the susceptibility and potential tropism of DENV-4 for liver, lung and heart of an immunocompetent mice model, and to evaluate and investigate the resulting morphological and ultrastructural alterations upon viral infection. BALB/c mice were inoculated intravenously with non-neuroadapted doses of DENV-4 isolated from a human case. The histopathological analysis of liver revealed typical alterations of DENV, such as microsteatosis, edema and vascular congestion, while in lung, widespread areas of hemorrhage and interstitial pneumonia were observed. While milder alterations were present in heart, characterized by limited hemorrhage and discrete presence of inflammatory infiltrate, the disorganization of the structure of the intercalated disc is of particular interest. DENV-4 RNA was detected in liver, lung, heart and serum of BALB/c mice through qRT-PCR, while the NS3 viral protein was observed in all of the aforementioned organs through immunohistochemistry. These findings indicate the susceptibility of the model to the serotype and further reinforce the usefulness of BALB/c mice in studying the many alterations caused by DENV.

## 1. Introduction

Dengue is a global emerging human disease, thriving in impoverished urban areas, suburbs and rural regions, but also present in wealthier areas of tropical and subtropical countries. The disease is caused by the dengue virus (DENV), an arbovirus transmitted by mosquitoes of the genus *Aedes*, composed of four distinct but antigenic-related serotypes: DENV-1, -2, -3 and -4 [1]. Dengue is present in at least 128 countries, with around 50 million to 200 million infections occurring every year [2]. While DENV-4 was first detected in Brazil in 1981–82 in the northern region of the country, its reintroduction did not occur until many years later in 2010 [3], causing outbreaks in most of the Brazilian states in the following years [4].

Characterized by a wide spectrum of clinical manifestations, dengue can have an unpredictable evolution. Manifestations vary from a self-limited fever with spontaneous resolution to much more severe cases of plasma leakage associated with hemorrhage. A vast majority of cases remain asymptomatic, without notable clinical signs [5,6]. When present, these signs appear after a period of 3 to 15 days [7].

Pathological alterations in liver are among the most commonly observed during DENV infections, being closely associated with severe dengue cases [8]. Human patients normally present jaundice, acute hepatitis, hepatomegaly, liver failure and elevated levels of serum albumin. Elevated levels of aspartate transaminase and alanine transaminase enzymes are commonly observed in dengue secondary infections [9,10,11,12,13]. Microscopically, many areas of vascular congestion, as well as many hemorrhagic foci can be observed. Several areas presenting inflammatory infiltrate can be observed close to the portal space, as well as focal vacuolization of the cytoplasm of hepatocytes, areas presenting hemorrhagic congestion, hyperplasia of Kupffer cells and presence of lipid inclusions within hepatocytes [10,14,15]. Ultrastructural alterations observed include micro and macrosteatosis, focal areas of necrosis, with mononuclear inflammatory infiltrate, mitochondrion ingurgitation, characteristic of the apoptotic process and areas of hemorrhage and edema [15].

BALB/c mice infected by intravenous and intraperitoneal routes with non-neuroadapted DENV-2, isolated from a human case, presented areas of congestion of the central and portal veins, diffuse steatosis, hypertrophy of Kupffer cells, inflammatory cells within the interstice, portal space and sinusoids, endothelial cells with cytoplasmic electrondense inclusions, edema and focal areas of necrosis, monocytes and lymphocytes in sinusoids and of lymphocytic infiltrate close to the portal space. Nuclear lipid-like inclusions in hepatocytes were also observed [16,17,18]. In a study of BALB/c mice infected with two distinct Brazilian lineages of DENV-2, Jácome et al. [19] showed livers with vascular congestion, presence of inflammatory infiltrate in the proximity of the portal area, hepatocyte ballooning degeneration, nuclear area enlargement, with chromatin pattern alterations and sinusoid capillary dilation around centrolobular veins and nuclear atypia. A singular case presented lipid droplets within the parenchyma, and signs of necrosis were observed in one of the analyzed lineages.

Pulmonary manifestations in dengue human cases are uncommon. However, alterations such as bronchial thickening, bilateral pulmonary congestion, pleural effusion, hemoptysis and pneumonitis have been reported [20,21]. More unusual manifestations include acute respiratory distress syndrome and pulmonary dysfunction [10]. Histopathological and ultrastructural analysis of the lung of dengue fatal cases revealed interstitial pneumonia, thickening of the alveolar septum, diffuse mononuclear and polymorphonuclear cell infiltrate, areas of alveolar congestion, formation of hyaline membrane, hyperplasia of type II pneumocytes, platelet recruitment, areas of hemorrhage and edema and presence of viral particles in the endothelium [14,15,16,22].

The analysis of lung tissue of BALB/c mice intravenously infected with non-neuroadapted DENV-2 samples revealed the presence of interstitial pneumonia, thickening of alveolar septum, alveolar macrophages and erythrocytes in the alveolar space, peribronchiolar infiltrate, platelet recruitment, mononuclear and polymorphonuclear cell infiltrate within the vases, endothelial cells emitting filopodia and focal areas of hemorrhage [17,23,24,25].

So far, the symptomatology and histopathology associated with DENV infection of the heart are not clearly understood [26]. Abnormal aspects of cardiac function observed in DENV infection include sinusoidal tachycardia, increased jugular pressure, alterations in the cardiac rhythm and interstitial edema [27], as well as bilateral systolic ventricular dysfunction, this last one being more commonly associated with cases of severe dengue [28]. Cases of myocarditis in DENV infections may remain asymptomatic through the course of the disease [29], although the condition is associated with severe clinical evolutions, such as hypotension, arrhythmia, circulatory collapse and cardiogenic shock [9,11,30,31,32,33]. X-rays of dengue human patients revealed a progressive case of cardiomegalia [21,34] and the histopathological analysis of the heart of fatal cases revealed nuclear and mitochondrial alterations, degeneration of cardiac fibers, hemorrhage, interstitial edema, diffuse inflammatory infiltrate and the presence of macrophages in the myocardium [10,26,35].

Jácome et al. [25] showed that the cardiac tissue of BALB/c mice infected with DENV-1 and reinfected four months later with either DENV-2 or -3 presented vascular congestion and mononuclear inflammatory cells and erythrocytes in the interstice. Additionally, ultrastructural analysis revealed the accumulation of liquid within the capillary, mitochondrial cristae degeneration, degranulated platelets adhered to the capillary wall and swollen endothelial cells, due to intense trafficking of vesicles between endothelial cells and cardiomyocytes. Virus-like particles were observed in the interstice between cardiomyocytes.

Currently, the mechanisms associated with the pathogenesis of DENV and the factors that determine the occurrence of severe dengue are not thoroughly understood. This is mostly due to the lack of a proper animal model that replicates the infection as it occurs in human patients [36,37,38,39]. The inexistence of an adequate animal model that reproduces the DENV infection spectrum is an issue constantly reported [40,41,42,43,44,45,46]. Many of the models suggested in the related literature utilize humanized or immunodeficient mice [47,48], invasive inoculation routes and neuroadapted viral strains [49,50,51]. Moreover, some studies reinforce the lower competence of immunocompetent models in presenting viremia when infected with DENV strains isolated from patients, with the exception of newborn animals and when the intracranial inoculation route is utilized [47,52,53]. Nevertheless, previous studies have shown that BALB/c mice infected with non-neuroadapted DENV-1, -2 and -3 strains produced from clinical isolates and inoculated via intraperitoneal or intravenous route presented viremia and tissue alterations similar to those described in dengue human cases [16,17,18,23,24,25,54,55]. 

Due to its low cost and easy handling, immunocompetent murine models, such as the C57BL/6 strain [56], A/J strain [57,58] and BALB/c strain [49,59,60,61,62], are generally considered as the most viable models for DENV experimental infection. However, a challenge for their validation so far has been their low susceptibility to DENV. Attempts at establishing immunocompetent models normally utilize invasive inoculation routes, such as the intracranial route, and inoculum dose of high viral titers (>10^9^ PFU), generally resulting in neurovirulent DENV infections [63,64,65].

Previous studies have shown that the experimental infection of immunocompetent BALB/c mice with a non-neuroadapted DENV-2 strain, by either intraperitoneal or intravenous routes, resulted in histopathological alterations similar to those commonly described in dengue human cases, with no fatalities observed in the mice [16,17,19,23,24].

Here, we aimed to investigate the susceptibility and the histopathological and ultrastructural alterations caused in immunocompetent adult BALB/c mice intravenously inoculated with a non-neuroadapted epidemic DENV-4 strain.

## 2. Materials and Methods

### 2.1. Ethics Statement

All the procedures performed in this study were approved by the Animal Ethics Committee of Oswaldo Cruz Institute (IOC), Oswaldo Cruz Foundation (Fiocruz) under the protocol number LW-50/11 and all experiments were performed in accordance with relevant guidelines and regulations.

### 2.2. Viral Strain

The DENV-4 strain used in this study (BR2972/2013) was isolated from a 24-year-old female patient living in the city of Niterói, Rio de Janeiro, Brazil, presenting fever, prostration, headache, retro-orbital pain, myalgia, nausea, vomiting, diarrhea, lombalgia and abdominal pain on 4 April 2013. The serum sample was collected 5 days after the onset of symptoms and presented a positive result by IgM ELISA, NS1 capture ELISA and Real Time Quantitative (q) RT-PCR [66]. DENV-4 was the infecting serotype identified by qRT-PCR and by isolation into *Aedes albopictus* cell line (C6/36 cells) [67] and indirect fluorescent antibody test using serotype specific monoclonal antibodies [68]. Case confirmation was performed by the Flavivirus Laboratory at the IOC and kindly provided for use in this study. There was no human involvement for the purpose of this study. Furthermore, the viral strain utilized was from the first passage after original isolation in cell culture and did not undergo any passage through mice brain, avoiding neuroadaptation.

### 2.3. Viral Stock Production

The DENV-4 viral stock was produced by inoculation in a 175 cm^2^ flask containing C6/36 cells in a concentration of 5 × 10^5^ cells/mL. After titration using the Reed and Muench method [69], the viral stock was kept at −80 °C until use. After three cell passages, the strain presented a viral titer of 10^9^ TCID_50_/mL, and was selected for experimental infection.

### 2.4. Animals

Two-month-old male BALB/c mice, weighing between 20 and 25 g, were obtained from the Institute of Science and Technology in Biomodels, at Fiocruz, Rio de Janeiro, Brazil. During the experimentation period, the animals were contained in cages attached to ventilated shelves, where conditions such as temperature, humidity, feeding, ventilation, hygiene and photoperiod were rigorously controlled. Upon arrival in the vivarium, mice were separated into three groups of five mice to ease handling and to avoid fighting. Physical and behavioral alterations, when present, were properly recorded. The number of mice used for each group is listed in Table 1.

### 2.5. Temperature Gauging and Clinical Aspects

The body temperature of every animal was verified pre-infection and post-infection (prior to euthanasia). The thermometer was embedded in mineral oil, gently inserted into the mice’s rectum for one minute and the temperature was properly recorded.

### 2.6. Statistical Analysis

A database on the temperatures post- and pre-infection was created in Microsoft Excel (Microsoft Corporation, Redmond, Washington, DC, USA) and data were analyzed using the GraphPad Prism software version 8.0.1 (Graphpad Software, San Diego, CA, USA). Statistical analysis was performed using the SPSS Statistics software version 25 (IBM, Armonk, New York, NY, USA). Results of *p* ≤ 0.05 were considered statistically significant.

### 2.7. Experimental Infection

During inoculation, mice were contained in an acrylic support with the tail remaining carefully stretched. A dose of 100 μL of the inoculum, presenting a titer of 10,000 TCID_50_/0.1 mL, was administered through the caudal vein using a disposable needle. Mice of the control group were inoculated with 100 μL of Leibovitz (L-15) culture medium (Sigma-Aldrich, Schnelldorf, Bavaria, Germany).

### 2.8. Euthanasia and Organ Excision

All the mice were euthanized 72 h post-inoculation. The anesthesia was performed using a lethal dose (150 mg/kg) of sodium thiopental (Thiopentax, Cristália, Itapira, São Paulo, Brazil), administered through the intraperitoneal route. Once the anesthetic effect set in, the blood was collected through cardiac puncture, and the mice were subject to cervical dislocation. Mice whose organs were destined to transmission electron microscopy analysis were subject to fixation through perfusion for dissection and organ excision. Organs were processed and properly stored according to the intended analysis technique.

### 2.9. Serum Sampling

Blood samples were collected from infected mice through cardiac puncture and stored in microtubes. A volume of 0.7 mL to 0.8 mL of blood was collected from each mouse, followed by the immediate euthanasia of the animal. The blood was then centrifuged in a refrigerated centrifuge (4 °C) for 10 min, at 5000 rpm, to separate the serum from the cellular components. Serum samples were stored at −80 °C for molecular analysis. As the technique used for blood collection is invasive and may damage the tissue, blood was collected from a different group of mice (Table 1) to avoid altering the integrity of the heart. For this reason, the results of serum molecular analysis could not be paired with other results.

### 2.10. Bright Field Microscopy

Organs destined to bright field microscopy were fixated in Millonig’s buffered formalin and stored in a refrigerator. The tissue samples were dehydrated in decreasing concentrations of ethanol, clarified in xylene and embedded in paraffin. Tissue sections 5 µm thick were obtained using a microtome and stained with hematoxylin and eosin for posterior analysis using a bright field microscope.

### 2.11. Fixation by Perfusion and Transmission Electron Microscopy

Mice whose organs were analyzed through transmission electron microscopy were subject to perfusion with a fixative solution (4% paraformaldehyde in sodium phosphate buffer 0.2 M, pH 7.2). Following the procedure, the organs were collected and fixated in 3% glutaraldehyde in sodium cacodylate buffer 0.2 M, pH 7.2. Afterwards, the organ fragments were washed with 0.2 M sodium cacodylate buffer in 7% sucrose, post-fixated in 2% aqueous osmium tetroxide and once again washed in 0.2 M sodium cacodylate buffer in 7% sucrose. Afterwards, the samples were dehydrated in acetone and finally embedded in epoxy resin [70]. The resulting hardened resin was sliced in ultrathin sections 50–70 nm thick with an ultramicrotome and placed on copper grids. The ultrathin sections were stained with uranyl acetate and lead citrate according to Reynolds [71] and analyzed using a transmission electron microscope (JEOL-JEM-1011, JEOL, Tokyo, Japan).

### 2.12. Immunohistochemistry

Glass slides containing paraffin-embedded samples of liver, lung and heart were heated at 60 °C, deparaffinized in xylene and rehydrated in ethanol. Antigen retrieval was performed by heating the tissue in a pressure cooker in the presence of EnVision Flex target retrieval solution, high pH (Agilent, Santa Clara, CA, USA). Endogenous peroxidase was blocked with hydrogen peroxidase in methanol (1:1). To reduce non-specific binding, samples were incubated for 10 min at room temperature using a protein blocker solution (Spring Bioscience, Pleasanton, CA, USA). The samples were then incubated overnight at 4 °C with anti-NS3 antibody produced in rabbit (1:200), produced in-house and provided by the Laboratory of Biotechnology and Structural Bioengineering of the Rio de Janeiro Federal University. Afterwards, the samples were incubated with anti-rabbit antibody horseradish peroxidase conjugate (Spring Bioscience, Pleasanton, CA, USA). Negative control samples were incubated only with the secondary horseradish peroxidase-conjugated antibody. Finally, the reaction was revealed with diaminobenzidine (Agilent, Santa Clara, CA, USA) as chromogen and sections were counterstained with Harris hematoxylin (Agilent, Santa Clara, CA, USA).

### 2.13. Molecular Biology

Organs for the molecular analysis were washed with phosphate buffered saline and stored in microtubes at −80 °C until use. Afterwards, 500 µL of L-15 culture medium (Invitrogen Corporation, Waltham, MA, USA) was added to the microtubes, samples were macerated using disposable plastic pestles and centrifuged for 15 min at 10,000 rpm at 4 °C. The extraction was performed using 140 µL of macerate supernatants and serum samples, using the QIAmp Viral RNA mini kit (Qiagen, Hilden, North-Rhine Westphalia, Germany) according to the protocol described by the manufacturer.

### 2.14. Real-Time Quantitative RT-PCR

The amplification performed using the SuperScript III Platinum One-Step Quantitative RT-PCR kit (Invitrogen Corporation, Waltham, MA, USA) according to the kit’s instructions and using the primers DENJ-4R (5′TCCACCTGAGACTCCTTCCA3′) and DENJ-4F (5′TTGTCCTAATGATGCTGGTCG3′) and probe DENJ-4P (6-FAM 5′TTCCTACTCCTACGCATCGATTCCG3′ BHQ-1), as described by Johnson et al. [66], with slight modifications in the thermocycling conditions. The mixture (final volume of 20 µL) was prepared with 1 µL of each primer at 50 µM; 12.5 µL of the reaction mixture 2× (0.4 µM of each dNTP and 6 µM of MgSO4); 0.5 µL of the enzyme SuperScript III RT; 3.5 µL of DNase/RNase free water; 1 µL of MgSO4 at 5 mM; and 0.75 µL of the probe at 9 µM and loaded into a 96-microwell optical microplate (PE Applied Biosystems, CA, USA). Five microliters of the extracted RNA were added and the reaction was carried out in a LineGene 9660 thermocycler (Bioer, Hangzhou, Zhejiang, China). Thermocycling conditions consisted of a reverse transcription at 50 °C for 15 min, enzyme activation at 95 °C for 15 min and 40 cycles at 95 °C for 15 s and 60 °C for 1 min. According to the protocol, a positive result is considered up to a cycle threshold (Ct) value of 36. For viral RNA quantification, the same protocol was used, and the results were compared to a DENV-4 RNA standard curve.

## 3. Results

### 3.1. Clinical Aspects

No deaths were reported among the experimentally infected mice, which were all properly euthanized. No neurological alterations, such as paralysis or blindness, or other clinical signs, such as petechiae, hemoptysis, tremors, diarrhea and melena, were observed between DENV-4 infection and euthanasia. Clinical signs or alterations were not observed in non-infected mice.

**Figure 1:**
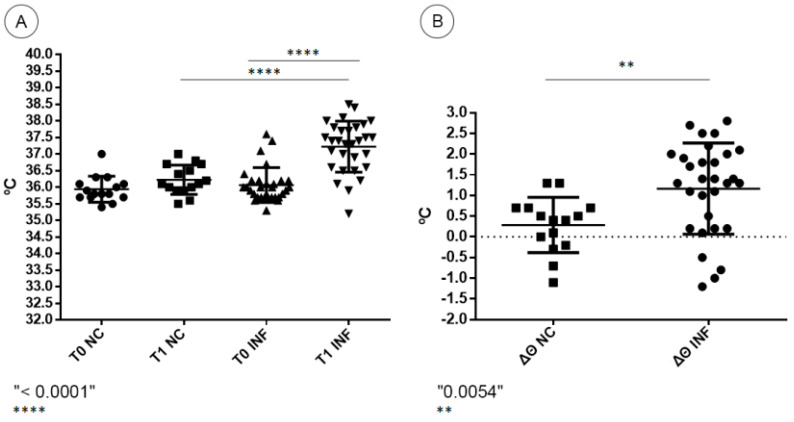
(**A**) Temperature values at pre-infection time (T0) and 72 h post-infection (T1) of negative control (NC) and DENV-4-infected BALB/c mice (INF). (**B**) Temperature variation between T0 and T1 (Δϴ) of negative control (NC) and DENV-4-infected BALB/c mice (INF).

A noteworthy increase in rectal temperature was observed in BALB/c mice infected with DENV-4, characterizing hyperthermia. Most infected animals presented an individual increase between the first (T0) and the second (T1) rectal temperature evaluations. The highest recorded value among infected mice was 38.5 °C. Negative control mice presented milder or no increase in rectal temperature, with a highest recorded temperature of 37 °C (Figure 1A). The mean increase in temperature in DENV-4-infected mice was statistically significant when compared to negative control mice (*p* < 0.0001). Regarding the temperature variation (Δϴ) between the two moments, negative control mice presented a mean increase of 0.28 °C, while infected mice presented a higher mean increase of 1.16 °C (Figure 1B). The temperature variation in DENV-4-infected mice was statistically significant when compared to negative control mice (*p* = 0.0054).

### 3.2. Morphological Analysis

**Figure 2:**
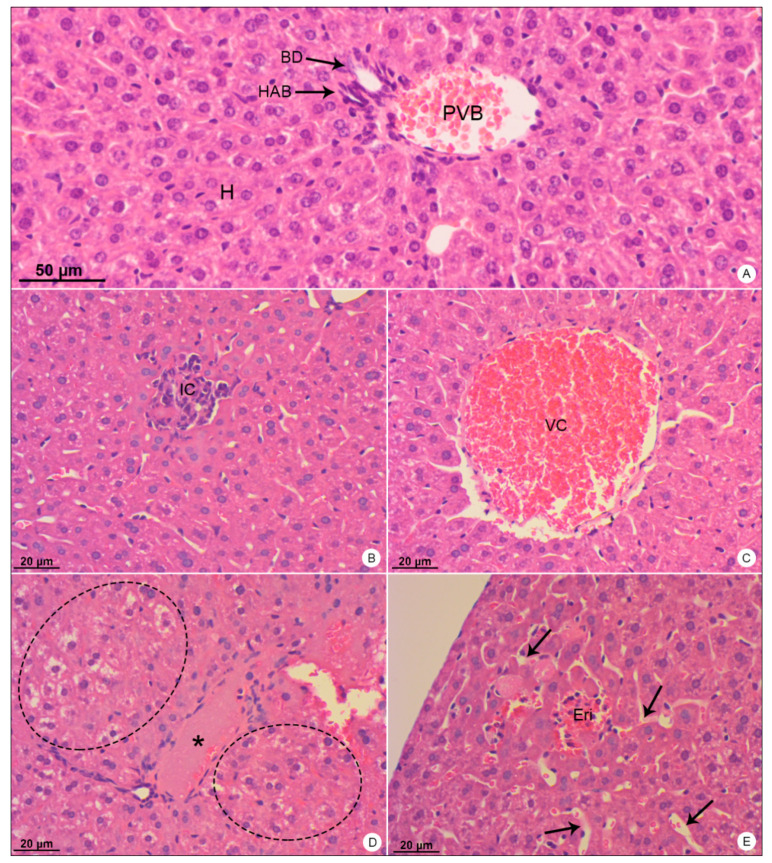
Liver samples of BALB/c mice stained with hematoxylin and eosin and observed in bright field microscope. (**A**) Negative control sample. Portal vein branch (PVB); hepatic artery branch (HAB); biliary duct (BD); hepatocytes (H). (**B**–**E**) DENV-4-infected samples. Inflammatory infiltrate (IC); vascular congestion (VC); edema (*); ballooning degeneration of hepatocytes (dashed outline); erythrocytes within the tissue (Eri); dilation of sinusoid capillaries (arrows). Magnification: (**A**) = 200×; (**B**) = 200×, (**C**) = 200×; (**D**) = 200×; (**E**) = 200×.

**Figure 3:**
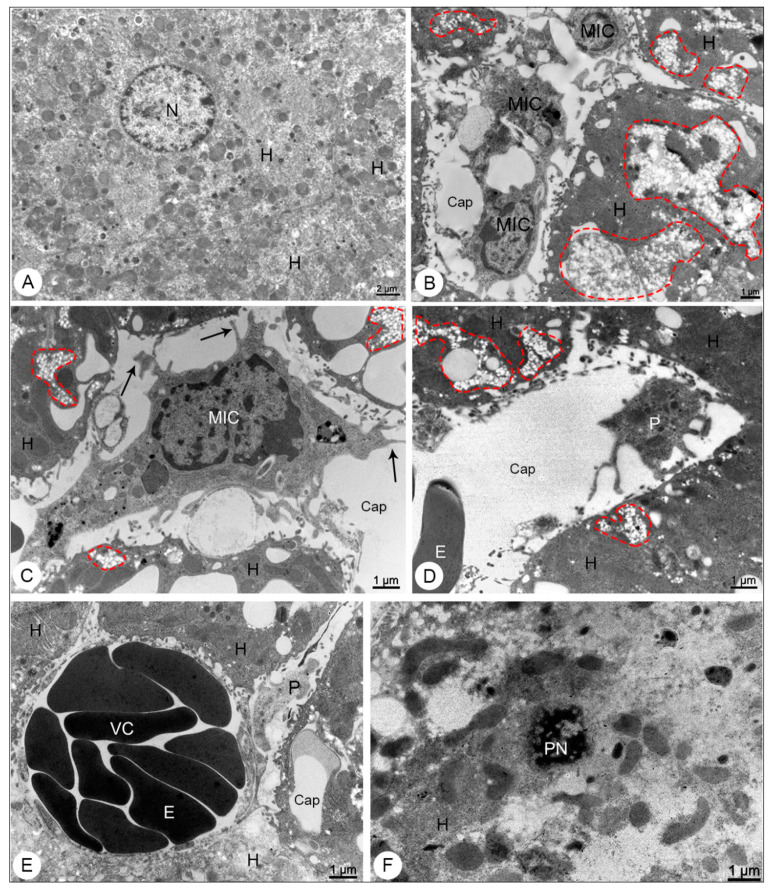
Liver samples of BALB/c mice observed in transmission electron microscope. (**A**) Negative control sample. Hepatocyte (H); nucleus (N). (**B**–**F**) DENV-4-infected samples. Microvesicular steatosis (dashed outline); mononuclear inflammatory cells (MIC) presenting filopodia (arrows); capillary (Cap); hepatocytes (H); activated platelet (P); erythrocyte; vascular congestion (VC); pyknoctic nucleus (PN). Magnification: (**A**) = 5000×; (**B**) = 8000×, (**C**) = 12,000×; (**D**) = 10,000×; (**E**) = 12,000×; (**F**) = 12,000×.

**Figure 4:**
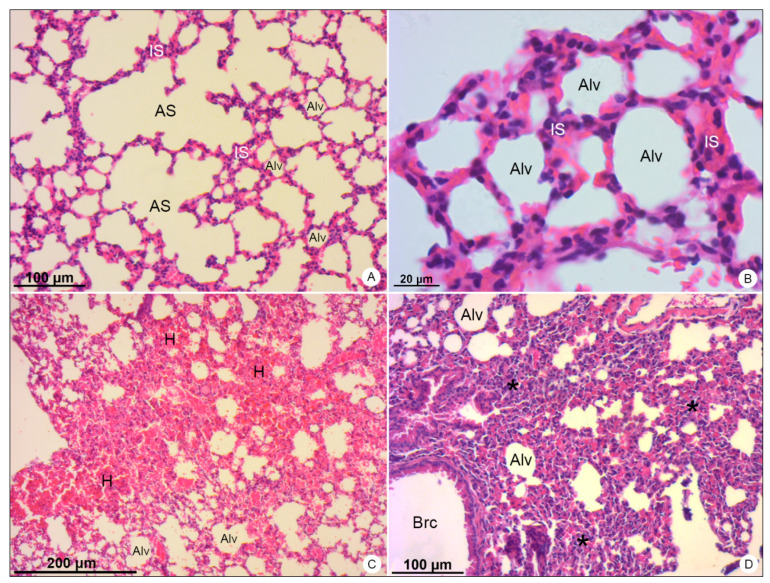
Lung samples of BALB/c mice stained with hematoxylin and eosin and observed in bright field microscope. (**A**,**B**) Negative control samples. Alveolar sac (AS); alveolus (Alv); interalveolar septum (IS). (**C**,**D**) DENV-4-infected samples. Areas of hemorrhage (H); thickening of the interalveolar septa (*); bronchiole (Brc). Magnification: (**A**) = 100×; (**B**) = 400×, (**C**) = 50×; (**D**) = 100×.

**Figure 5:**
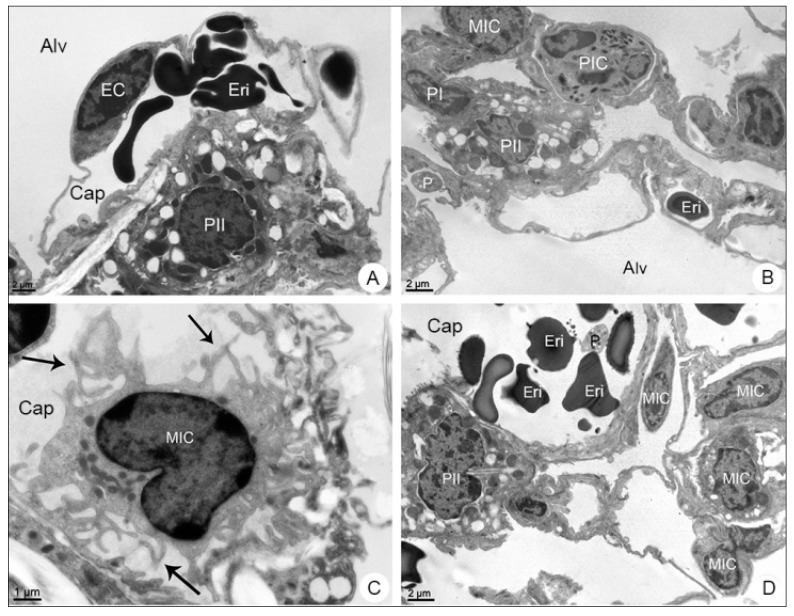
Lung samples of BALB/c mice observed in transmission electron microscope. (**A**) Negative control sample. Type II pneumocyte (PII); endothelial cell (EC); alveolus (Alv); capillary (Cap); erythrocyte (Eri). (**B**–**D**) DENV-4-infected samples. Type I Pneumocyte (PI); mononuclear inflammatory cell (MIC); polymorphonuclear cell (PIC); platelet (P); filopodia (arrows). Magnification: (**A**) = 6000×; (**B**) = 6000×, (**C**) = 12,000×; (**D**) = 6000×.

**Figure 6:**
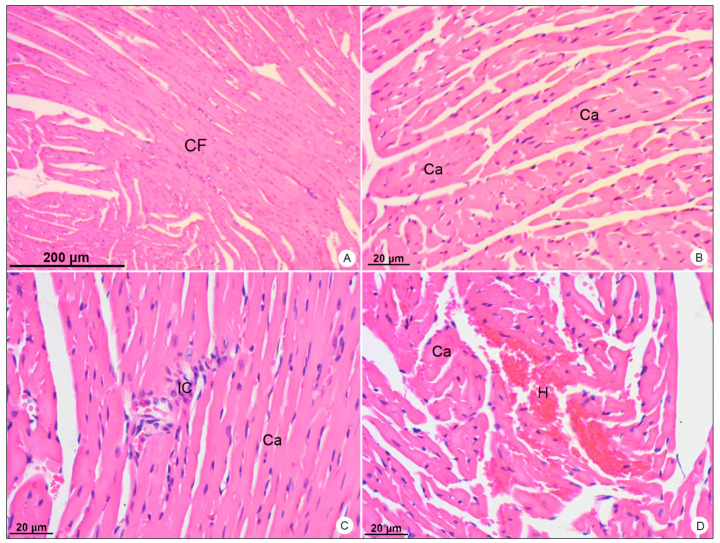
Heart samples of BALB/c mice stained with hematoxylin and eosin and observed in bright field microscope. (**A**,**B**) Negative control samples. Cardiac Fibers (CF); cardiomyocytes (Ca). (**C**,**D**) DENV-4-infected samples. Inflammatory infiltrate (IC); area of hemorrhage (H). Magnification: (**A**) = 50×; (**B**) = 200×, (**C**) = 200×; (**D**) = 200×.

**Figure 7:**
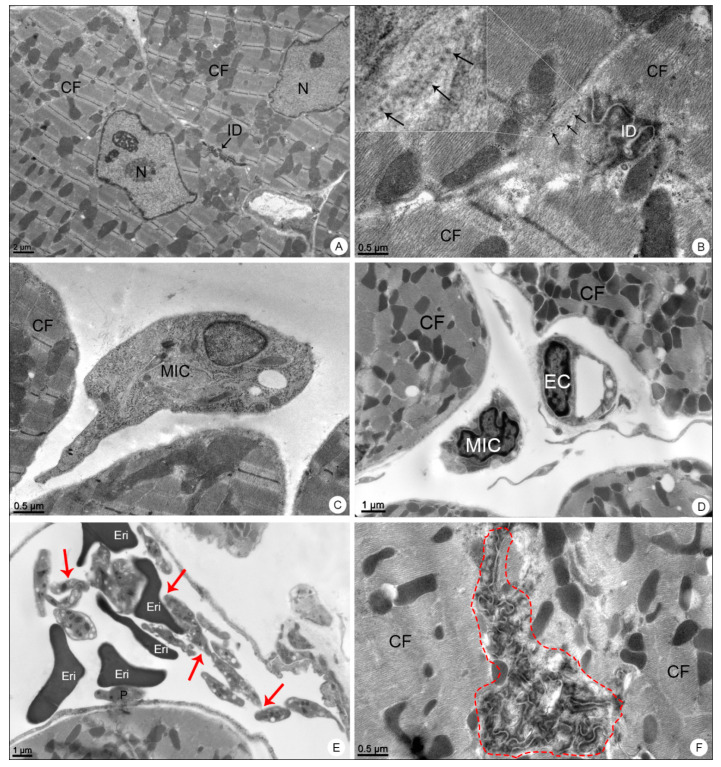
Heart samples of BALB/c mice observed in transmission electron microscope. (**A**) Negative control sample. Cardiac fibers (CF); nucleus (N); intercalated disc (ID). (**B**–**F**) DENV-4-infected samples. Dengue virus-like particles (arrows); intercalated disc (ID); mononuclear inflammatory cell (MIC); platelet aggregation (red arrows); platelet adhered to the erythrocyte (E), endothelium (P); capillary (C); intercalated disk disorganization (dashed outline). Magnification: (**A**) = 5000×; (**B**) = 30,000×, (**C**) = 15,000×; (**D**) = 12,000×; (**E**) = 10,000×; (**F**) = 20,000×.

Mice used as negative control showed no signs of histopathological or ultrastructural alterations. Tissue integrity and cell structure were well preserved in all organs analyzed (Figure 2A, Figure 3A, Figure 4A,B, Figure 5A, Figure 6A,B and Figure 7A).

#### 3.2.1. Liver Samples

Liver samples of DENV-4-infected mice showed inflammatory infiltrate (Figure 2B), signs of portal congestion (Figure 2C), presence of edema (Figure 2D), hepatocyte ballooning (Figure 2D), dilation of sinusoid capillaries (Figure 2E) and focal areas of hemorrhage (Figure 2E).

By transmission electron microscope, the liver samples of DENV-4 experimentally infected mice showed microvesicular steatosis (Figure 3B–D), mononuclear inflammatory cells presenting filopodia (Figure 3B,C), activated platelets within the capillary (Figure 3D,E), areas of vascular congestion (Figure 3E) and focal points of necrosis (Figure 3F).

#### 3.2.2. Lung Samples

In lung samples of DENV-4-infected mice, wide areas of hemorrhage (Figure 4C) were observed, as well as the thickening of alveolar septum, due to the migration of inflammatory cells to the pulmonary parenchyma (Figure 4D).

The transmission electron microscope analysis revealed the presence of mononuclear (Figure 5B–D) and polymorphonuclear (Figure 5B,D) inflammatory cells inside the capillary, as well as platelets and erythrocytes within the alveolar space (Figure 5D).

#### 3.2.3. Heart Samples

In heart samples of DENV-4-infected BALB/c mice, the presence of inflammatory infiltrate (Figure 6C) was observed, as well as focal areas of hemorrhage (Figure 6D).

When analyzed in transmission electron microscope, these samples showed the presence of DENV-like particles in cardiomyocytes (Figure 7B), mononuclear inflammatory cells (Figure 7C,D), platelet aggregation within the capillary and platelet adherence to the endothelium (Figure 7E) and alterations in the morphology of the intercalated discs (Figure 7F).

### 3.3. Immunohistochemistry

**Figure 8:**
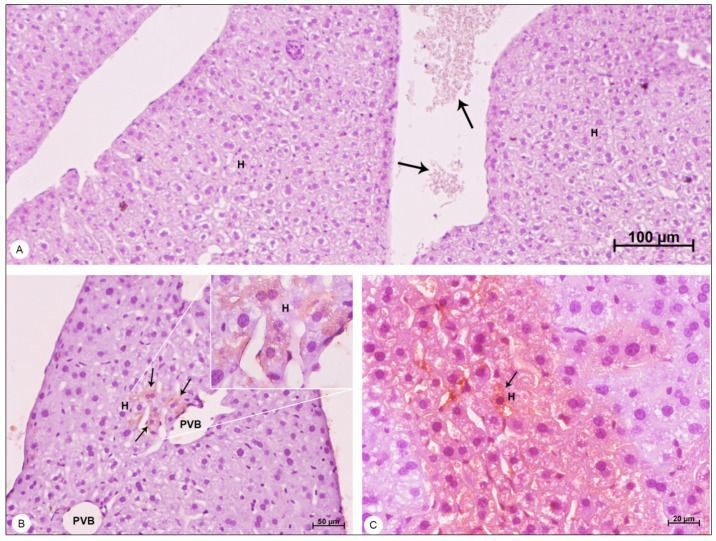
Liver samples of BALB/c mice counterstained with Harris hematoxylin and observed in bright field microscope. (**A**) Negative control sample. Hepatocytes (H); erythrocytes showing no peroxidase reaction (arrows). (**B**,**C**) DENV-4-infected samples. Portal vein branch (PVB); hepatocytes (H); areas of DENV-4 antigen detection (arrows). Magnification (**A**) = 100×; (**B**) = 200×, (**C**) = 400×.

**Figure 9:**
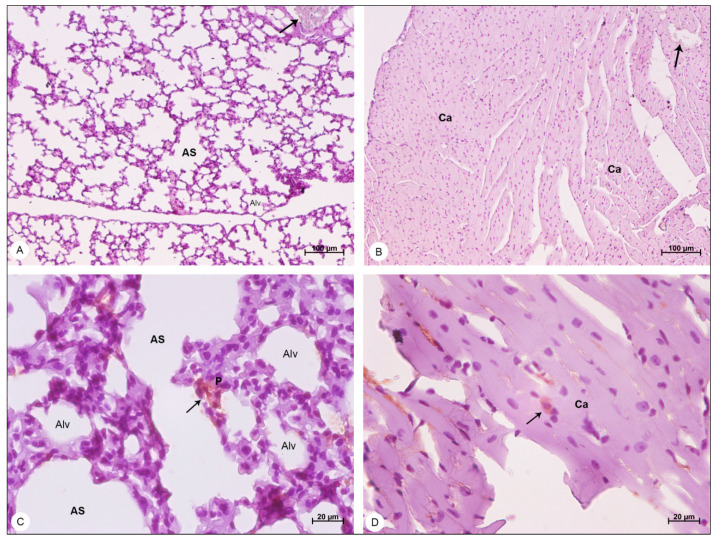
Lung and heart samples of BALB/c mice, counterstained with Harris hematoxylin and observed in bright field microscope, showing detection of the NS3 protein. (**A**) Negative control lung sample. Alveolar sac (AS); alveolus (Alv); erythrocytes showing no peroxidase reaction (arrow). (**B**) Negative control heart sample. Cardiomyocyte (Ca); erythrocytes showing no peroxidase reaction (arrows). (**C**) DENV-4-infected lung sample. Pneumocytes (P); areas of DENV-4 antigen detection (arrow). (**D**) DENV-4-infected heart sample. Areas of DENV-4 antigen detection (arrow). Magnification (**A**) = 50×; (**B**) = 50×, (**C**) = 400×; (**D**) = 400×.

Liver, lung and heart samples of DENV-4-infected mice and control mice were analyzed through immunohistochemistry for the presence of the NS3 viral protein. The control samples showed no signs of NS3 detection, presenting peroxidase-unreactive hepatocytes (Figure 8A), pneumocytes (Figure 9A), cardiomyocytes (Figure 9B) and erythrocytes (Figure 8A and Figure 9A,B).

The NS3 antigen was detected in hepatocytes of the liver (Figure 8B,C), in pneumocytes of the lung (Figure 9C) and in cardiomyocytes of the heart (Figure 9D).

### 3.4. Molecular Analysis

**Figure 10:**
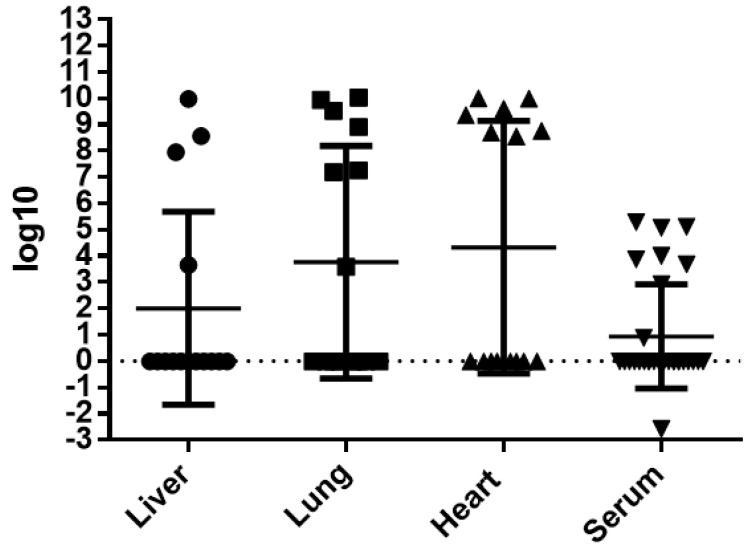
Individual titers of DENV-4 RNA detected in liver, lung, heart and serum of infected BALB/c mice, expressed in logarithmic values.

DENV-4 RNA was detected in 80% (12/15) of the infected mice, in at least one organ. Overall, viral RNA was detected in 43.9% (18/41) of all tissues analyzed and 30% (9/30) of the serum samples collected. The virus was more frequently detected in lung (46.6%; 7/15) and in heart (46.6%; 7/15) than in liver (36.4%; 4/11). The RNA titer detected in each individual organ or fluid, which ranged from 10^−3^ to 10^10^, is shown in Figure 10. The molecular analysis of liver, lung and heart pertain to the same group of mice, while the serum analysis was performed on a different group of mice to avoid heart alterations induced by the employed serum sampling technique, as previously stated.

## 4. Discussion

DENV-4 cases are normally characterized by milder symptoms and are less associated with hospitalizations, especially when compared to other serotypes [72]. However, factors such as secondary infection by heterologous DENV serotypes [73] and the interaction between dengue and other comorbidities may result in more severe disease and death [11]. Thus far, DENV-4 remains the least studied of the DENV serotypes, and data in the literature surrounding its histopathology and tropism are scarce.

In our analysis, a number of the infected mice presented an increase in the rectal temperature three days after inoculation. The body temperature of mice is described as unstable and variable, highly dependent on outside factors, such as strain, age, sex and environmental conditions [74]. Even so, a baseline temperature value of 36.9 °C is suggested [75]. Overall, this increase in mice temperature, or hyperthermia, can be associated with physiological or metabolic alterations in the organism of the mice, undetected in conditions of non-infection. This sign alone suggests the presence of an altered clinical condition, similar to what is observed in dengue human cases. Nevertheless, a decisive diagnosis of fever is impossible to achieve in mice, as it would require the detection of symptoms. The absence of an increase in temperature in some DENV infected mice could be explained by asymptomatic cases of dengue or a prolonged incubation period.

In this study, the liver of DENV-4-infected BALB/c mice revealed large caliber blood vessels presenting congestion, areas with mononuclear inflammatory infiltrate (particularly close to the portal space), focal areas of hemorrhage, areas of edema and ballooning degeneration of hepatocytes. The presence of activated mononuclear inflammatory cells was observed in the capillary and interstice, as well as areas of microvesicular steatosis. Sites of vascular congestion were also observed in smaller blood vessels, with the complete obstruction of capillary lumina. Focal points of necrosis were detected, characterized by the hepatocyte nucleus undergoing pyknosis. Activated platelets were observed within the capillary, suggesting alterations of blood vessel permeability, as previously described by Paes et al. [16], Barth et al. [17], Caldas [18] and Jácome et al. [19]. The alterations observed here correspond to those described in human fatal cases, with similar profiles of hemorrhage, inflammation and vascular congestion [9,11,12,14]. Even though the electron transmission microscope analysis did not show virus particles in the tissue, the DENV RNA was still detected in mice liver.

DENV-4 could possibly have a weaker tropism for mice liver cells, since the alterations observed seem to be much milder than those described during DENV-1, -2 and -3 infections. Steatosis, a hallmark of DENV liver infection in humans, was not widely observed in mice, being more noticeable on the ultrastructural level. Similarly, ballooning degeneration of hepatocytes, although present, was also not as widespread as is commonly reported in the literature. Lipid inclusions have been shown to play a major role in flavivirus replication [76], and the absence of ample lipid autophagy in hepatocytes could be associated with the detection of DENV RNA in fewer mice liver samples. Necrosis was also not widely observed here, nor was the presence of inflammatory infiltrate, which was discrete, and mostly observed in the midzonal areas of the liver, further away from the periportal region, where it is usually observed, alongside areas of necrosis [16,61,77]. Even so, the presence of vascular congestion, edema and hemorrhage could be associated to endothelium permeability alterations [78], further reinforced by the presence of activated platelets within the capillaries. Additionally, it has been previously suggested that during DENV infection, activated platelets are able to produce pro-inflammatory cytokines and have an important role in promoting plasma leakage [79]. Plasma leakage has also been associated with the interaction between the NS1 viral protein and toll-like receptor 4, which, in turn, also causes the activation of platelets, leading to thrombocytopenia through an enhancement of platelet aggregation [80].

In lung, mononuclear and polymorphonuclear inflammatory infiltrate in the tissue, thickening of the alveolar septum and areas of hemorrhage in the pulmonary parenchyma and in the alveolar space were observed. These findings are in agreement with those described in BALB/c mice by Barth et al. [17] and Barreto et al. [23,24], and present a similar profile to those described in human fatal cases. Alterations such as formation of hyaline membrane and presence of edema were not observed, nor was hyperplasia of type II pneumocytes, which presented a regular aspect. Although the presence of virus particles was not observed in lung, the viral genome was detected.

Due to the close association between the cardiovascular and pulmonary systems, the alterations observed during human DENV infections are particularly interesting. The thickening of the alveolar septum is caused by the presence of inflammatory cells in the pulmonary interstice [24], more closely observed through transmission electron microscopy. Furthermore, the extensive areas of hemorrhage might be associated with severe vascular permeability alterations [81]—likely induced by the NS1 viral protein [82]—or even to pulmonary hemorrhagic syndrome [83]. Even though vast areas of the respiratory surface were overtaken by hemorrhage and reduced due to the thickened septa, the mice did not present clinical signs such as hemoptysis or abnormal breathing, which suggests a self-limited lung involvement.

The alterations observed in heart of infected BALB/c mice in this study include intense platelet activity and many focal points of hemorrhage. As previously stated, the aggregation of platelets within the capillary may be induced by the presence of the NS1 viral protein. NS1-activated human platelets are also more likely to adhere to the endothelium and promote plasma leakage [80]. The presence of inflammatory infiltrate in the tissue suggests a mild case of myocarditis, a result of either the viral infection of cardiomyocytes [31], or of the action of pro-inflammatory cytokines on the heart’s tissue [84] (or both). Moreover, the presence of virus-like particles was observed in heart, among cardiac fibers and in close proximity to the intercalated discs, although not in endothelial cells. These results corroborate the previous findings described by Caldas [18], Jácome et al. [25] and Rasinhas [54]. However, alterations such as cytoplasm rarefaction and disorganization of the cardiac fibers were not observed in this study. Due to the association of cases of myocarditis to conduction disorders and arrhythmia, the inflammation of the myocardium could be connected to alterations observed in the morphology of the heart, as well as the intercalated discs, essential communicating junctions in the maintenance of a healthy cardiac contraction [85,86].

DENV-2 antigens in infected BALB/c mice have been previously shown in liver, a susceptible viral replication site, specifically in hepatocytes, endothelial cells and in hyperplasic Kupffer cells [16,17,87]. In humans, the detection of the DENV-3 NS3 protein has been shown in liver (in hepatocytes, endothelial cells and Kupffer cells), in lung (in alveolar macrophages, type II pneumocytes and endothelium) and in heart (in cardiac fibers, endothelium and monocytes) [8,88]. In this study, the NS3 viral protein was detected in hepatocytes, pneumocytes and cardiomyocytes, demonstrating the presence of the virus in liver, lung and heart and suggesting viral replication, which would only be possible in the presence of the NS3 protein [15].

DENV RNA was detected by RT-PCR and qRT-PCR in the serum, spleen, liver, lung, heart, brain, kidney and lymph node of human infected patients [89,90,91,92,93,94] and in blood extracted directly from the heart of a fatal case [92]. Moreover, the detection of DENV in a fatal human case, and the observation of virus-like particles in spleen, liver, heart and lung was described by Limonta et al. [95].

In BALB/c mice, Paes et al. [16] detected DENV RNA in liver and in serum, as well as viral antigens in hepatocytes and hepatic endothelial cells. The peak of viremia was observed 7 days post-infection with up to a tenfold increase in the number of infected cells. DENV RNA in infected BALB/c has also been described by Caldas [18] in spleen, cerebellum, liver, lung and kidney; by Rasinhas [54] in heart and serum; and by Jácome et al. [19] in liver. 

In this study, viremia in the infected mice ranged from 10^3^ to 10^5^ copies of RNA/mL. In three out of four livers, viral titers were above 10^7^ copies of RNA/mL. High titers, varying from 10^8^ to 10^10^ copies of RNA/mL, were observed in 7 out of 15 heart samples. Likewise, high titers were observed in 6 out of 15 tested lung samples, with a single sample presenting a titer of 10^3^ copies of RNA/mL. In some animals, the presence of viral RNA in more than one organ or fluid was observed. It is worth noting that in two animals, the presence of DENV-4 RNA and NS3 antigen was detected in heart and lung.

## 5. Conclusions

Ultimately, immunocompetent BALB/c mice intravenously infected with a non-neuroadapted epidemic DENV-4 strain isolated from a human case presented histopathological and ultrastructural alterations similar to those observed in dengue human patients, and DENV RNA was detected in liver, lung, heart and serum. Furthermore, the detection of the NS3 viral protein in those organs confirms not only the presence of the virus in the tissue, but also suggests its replication within the cells. Such findings indicate the susceptibility of BALB/c to DENV-4 infection, due to its capability of reproducing tissue alterations analogous to those observed in humans and to the production of viremia and replication in different tissues. Particular results, such as a high detection rate and high titers of DENV RNA observed in heart, and alterations in the intercalated disc’s morphology—findings not commonly described in DENV infection of heart—suggest that DENV-4 could have a stronger tropism to this organ, at least in the murine model. Moreover, the disruption of the integrity of the intercalated disc could explain cases of cardiac rhythm dysfunctions in humans, still not fully understood. Unfortunately, the results of transmission electron microscopy and bright field microscopy could not be paired, due to the possibility of damaging the heart during cardiac puncture. This limits the comparative potential of this study, as histopathological and ultrastructural results come from different animals. Overall, data on DENV-4 and its role during infection are still scarce, and many of the histopathological and ultrastructural alterations caused by it are yet to be uncovered. 

## Figures and Tables

**Table 1:** Number of BALB/c used for histopathology, immunohistochemistry and molecular analysis, transmission electron microscopy, viremia analysis and negative control.

N = 75	Histopathology/IHQ/qRT-PCR	TEM	Viremia
DENV-4 72 h.p.i.	15	15	30
Negative Control	10	5	

IHQ: immunohistochemistry, TEM: transmission electron microscopy, h.p.i.: hours post-infection.

## Data Availability

Not applicable.
